# Two new species of Hydromedusae from Queensland, Australia (Hydrozoa, Leptothecata)

**DOI:** 10.3897/zookeys.783.26862

**Published:** 2018-09-03

**Authors:** Julian Uribe-Palomino, Sarah Pausina, Lisa-ann Gershwin

**Affiliations:** 1 CSIRO Oceans & Atmosphere, Queensland Bioscience Precinct (80), 306 Carmody Road, St Lucia, Brisbane, Queensland, Australia 4067 CSIRO Oceans & Atmosphere Brisbane Australia; 2 School of Biological Sciences, University of Queensland, St Lucia, Queensland, Australia 4072 University of Queensland St. Lucia Australia; 3 CSIRO Oceans & Atmosphere, Castray Esplanade, Hobart, Tasmania, Australia 7000 CSIRO Oceans & Atmosphere Hobart Australia

**Keywords:** coastal ecology, endemic, gelatinous zooplankton, Great Barrier Reef, IMOS, Laodiceidae, Lovenellidae, Medusozoa, systematics, taxonomy

## Abstract

Two new species of small hydromedusae were found during routine monitoring in coastal waters of eastern Australia and are here described. The first, *Melicertissaantrichardsoni* Uribe-Palomino & Gershwin, **sp. n.**, from Moreton Bay, Queensland, is placed in its genus because of its possession of both cordyli and eight-fold symmetry. It differs from its congeners in two conspicuous features: firstly, having small, oval split gonads located adjacent to the base of the stomach, and secondly, in its extremely small size at maturity (2 mm bell diameter, compared to the next smallest species at 7 mm). Moreover, it possesses a unique combination of other characters. This species appears to be endemic to Moreton Bay. The second new species, *Paraloveniayongalensis* Gershwin & Uribe-Palomino, **sp. n.**, from the Great Barrier Reef, Queensland, is placed in its genus because of its two opposite normal tentacles and two opposite marginal clusters of cirri. It differs from its congeners primarily in a more rounded body than the others; the shape, length, and position of its short spindle-shaped, distal gonads; possession of subumbrellar nematocyst clusters; and possession of statocysts. These discoveries bring the total number of *Melicertissa* species to eight and the total number of *Paralovenia* species to three. The discovery of these two micromedusae underscores the need for further examination of the often-ignored minute and/or gelatinous fauna.

## Introduction

Increasing global scientific attention on jellyfish has focused mostly on large and conspicuous species; however, approximately 90% of jellyfish species are small and rarely noticed (Gershwin 2013). Only one longitudinal study on small medusae has been published, finding a five-fold increase in biomass and a complete shift in species dominance over a 20 year period in Jiaozhou Bay, China (Sun et al. 2012), suggesting that they might be more important ecologically than previously appreciated. Elsewhere, however, these smaller species have been virtually ignored.

Around Australia, gelatinous zooplankton are now routinely monitored along with other plankton, under the umbrella of the Australian Plankton Survey, a collaboration between the Integrated Marine Observing System (IMOS) and the Commonwealth Scientific Industrial Research Organisation (CSIRO). Four recent sampling programs under this umbrella have collected numerous specimens comprising two species of hydromedusae new to science.

**Figure 1.**
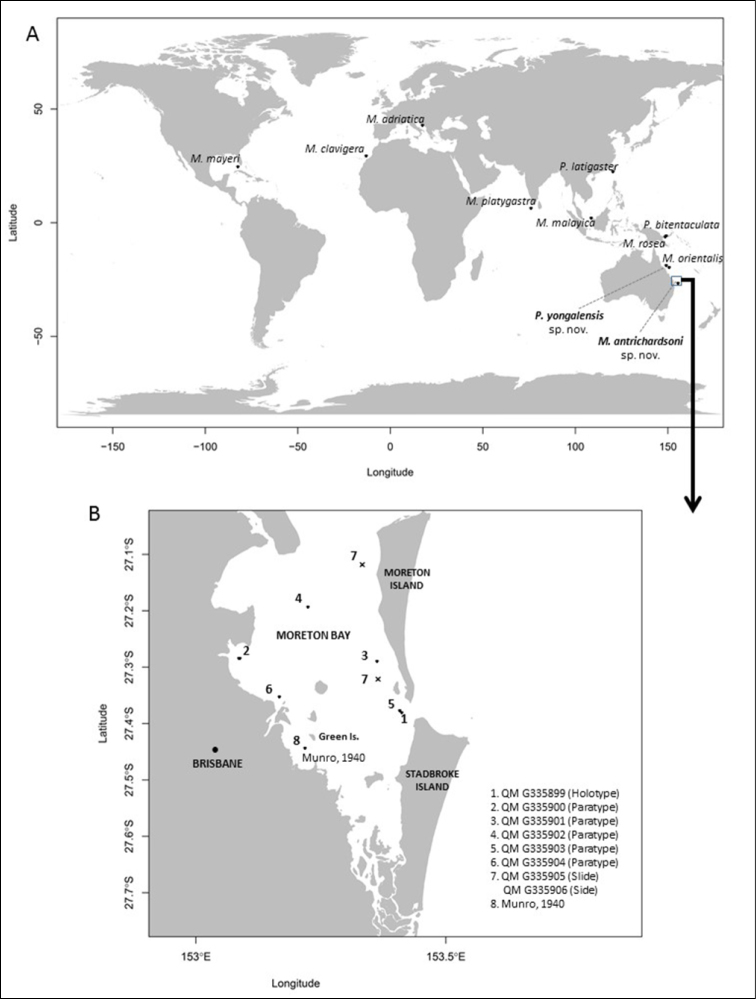
**A** Distribution of type localities of species of *Melicertissa* and *Paralovenia***B** Locations in Moreton Bay, Queensland, Australia, where *M.antrichardsoni* sp. n. was collected.

Seven species of the genus *Melicertissa* have been described since it was proposed by Haeckel in 1879. Five of those species have been found in tropical and subtropical waters of the northern hemisphere. The remaining two species, *M.orientalis* Kramp (1961) and *M.rosea*Bouillon (1984), were both found in the tropics of the southern hemisphere, in coastal waters of the Great Barrier Reef (GBR) and Papua New Guinea (PNG) respectively. *Paralovenia* is another genus of micromedusa with only two species recorded in the Western Pacific, *P.latigaster* Xu & Huang (2004), from the waters of the Taiwan Strait and *P.bitentaculata*Bouillon (1984), from PNG (Figure 1).

Here, we describe two new species from Queensland coastal waters, the first, *Melicertissaantrichardsoni* sp. n., is based on a few specimens collected in Moreton Bay, and the second, *Paraloveniayongalensis* sp. n., is based on a single specimen from tropical waters of the GBR. Both new species constitute the most southerly record for their respective genus. Neither of these species has been found in any other area of Australia or the world, suggesting endemism to eastern Australian waters. The purpose of this paper is to describe these two new species, thus adding to our baseline knowledge of Australia’s marine biodiversity.

## Materials and methods

### Specimen collection

Specimens were collected as part of four separate studies in Queensland, Australia, three in the subtropics and one in the tropics. The locations where the specimens were found and the distribution of their congeners is shown in Figure 1A, B.

Study 1: University of Queensland PhD project (Sarah Pausina), four specimens December 2010 to April 2011. Specimens were collected as part of a study on the zooplankton community dynamics within Moreton Bay, a shallow subtropical embayment. Stations along a salinity and nutrient gradient were sampled from the eutrophic Brisbane River mouth to the oligotrophic, oceanic-influenced Rous Channel. A 0.2 m mouth diameter, 100 µm mesh net fitted with a non-filtering cod-end (0.75 L) was towed horizontally within 1 m of the surface at approximately 1 m s^-1^ for 3-5 minutes during daylight hours.

Study 2: CSIRO, 3 specimens, March to December 2011. Specimens were collected as part of a project to evaluate the impact of the January 2011 Brisbane floods on the Moreton Bay area. Samples were collected every three months for one year from 13 sites around Moreton Bay, using a conical net with a 0.5 m diameter mouth and 100 μm mesh. The net was towed for 2 minutes at 1.5–2 knots following a linear transect collecting samples from the surface to 2 m deep.

Study 3: IMOS and CSIRO, 3 specimens, May 2013 and April 2014. Qualitative samples were collected as a supplement to the Moreton Bay plankton monitoring project. These samples were collected from Rous Channel in eastern Moreton Bay using a conical net of 0.5 m mouth diameter and 140 μm mesh. The net was towed at 1.5–2 knots following a linear transect collecting samples from the surface to 2 m deep.

Study 4: IMOS and CSIRO, 1 specimen, September 2016. The specimen was collected during routine monitoring of zooplankton at the IMOS National Reference Station near the *SS Yongala* shipwreck site, south-east of Townsville, Queensland, Australia. The sample was collected during a vertical tow descending from the surface to 28 m depth, using a conical net with a mouth diameter of 0.6 m and 100 μm mesh.

Samples collected from the different projects were fixed and preserved in 10% formalin.

### Microscopic study

All specimens were examined in a preserved state. Bulk samples were sorted under a Leica M165C stereo microscope. Specimens were photographed with a Canon EOS-5D camera adapted to the microscope by a 2.5x Leica optical tube. High quality images of the specimens were produced by stacking multiple pictures from different depth planes with Helicon focus 6.7.1 software.

Nematocyst slides from *M.antrichardsoni* sp. n. were prepared as described in Gershwin (2006) using Glycergel mounting medium, examined under a compound microscope with a 63x objective, and photographed with an iPhone 6s Plus held to the lens.

### Additional methods

Distribution maps were produced using the packages ‘map’ and ‘mapdata’ for the statistical computing and graphics software R 3.3.3 (R-Project) and the application RStudio 1.1.136 (RStudio).

Non-English descriptions were converted to text with OnlineOCR (https://www.onlineocr.net/) and translated online with Google Translate (https://translate.google.com.au/). Abbreviations used in the text: Bell diameter (BD) and stomach diameter (SD).

## Systematics

### Order LEPTOTHECATA Cornelius, 1992

#### Family LAODICEIDAE Agassiz, 1862

##### Genus *Melicertissa* Haeckel, 1879

###### Melicertissa antrichardsoni

Taxon classificationAnimaliaLeptothecataLaodiceidae

Uribe-Palomino & Gershwin sp. n.

http://zoobank.org/0DAF2DB1-E865-441B-885E-7A2031F320EA

Figures 2 , 3 , 4

####### Synonymy.

?*Melicertiasa* Haeckel, 1879 [incorrect spelling of Haeckel’s genus]: Munro 1940: 74. No figures or tables related to this specimen.

####### Type material.

**Figure 2.**
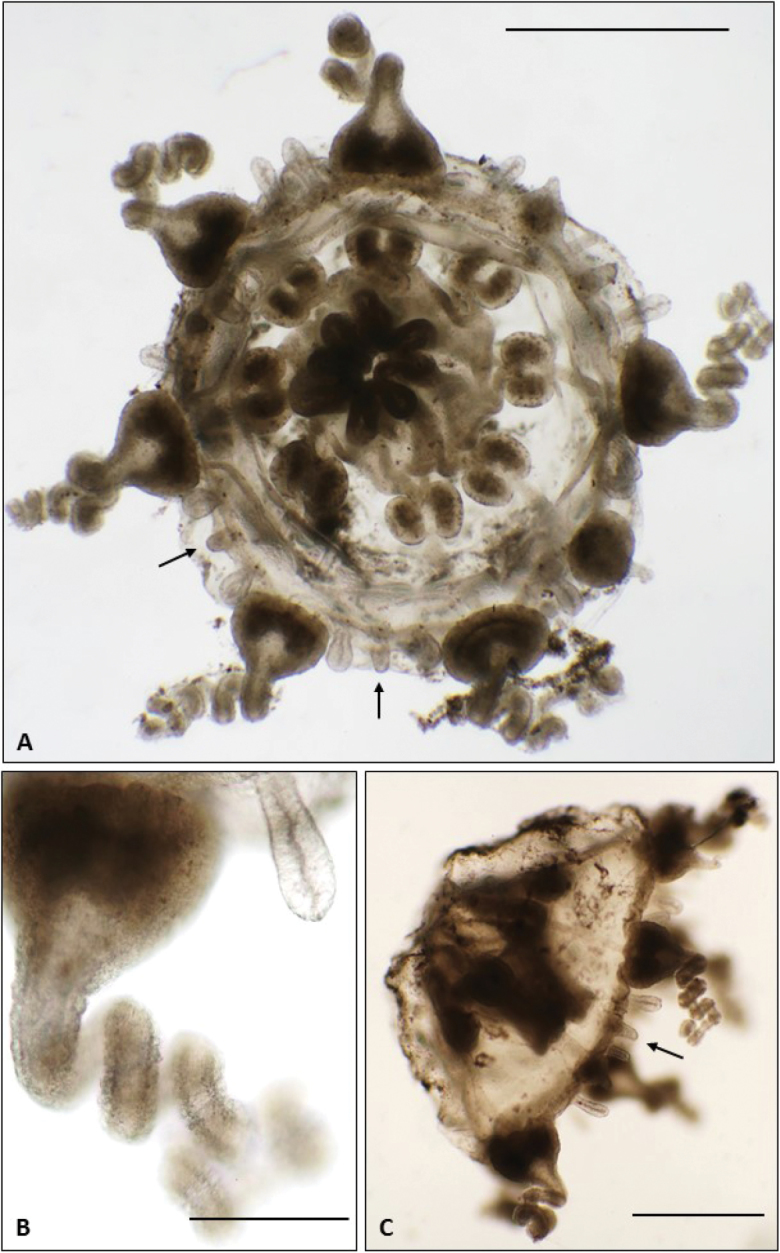
*Melicertissaantrichardsoni* sp. n. Holotype QM G335899. **A** Habitus, ventral view **B** Detail of tentacle and cordylis **C** Lateral view. Scale bars: 1 mm (**A, C**); 200 µm (**B**).

***Holotype***: QM G335899, Male, BD ca. 2 mm, tentacles six well developed and two rudimentary (Figures 2A, B, C; 4B, C); Moreton Bay, Queensland, Australia, 27.38°S, 153.39°E, 1 m, (Study 1), coll. S. Pausina, 15 Feb 2011.

**Figure 3.**
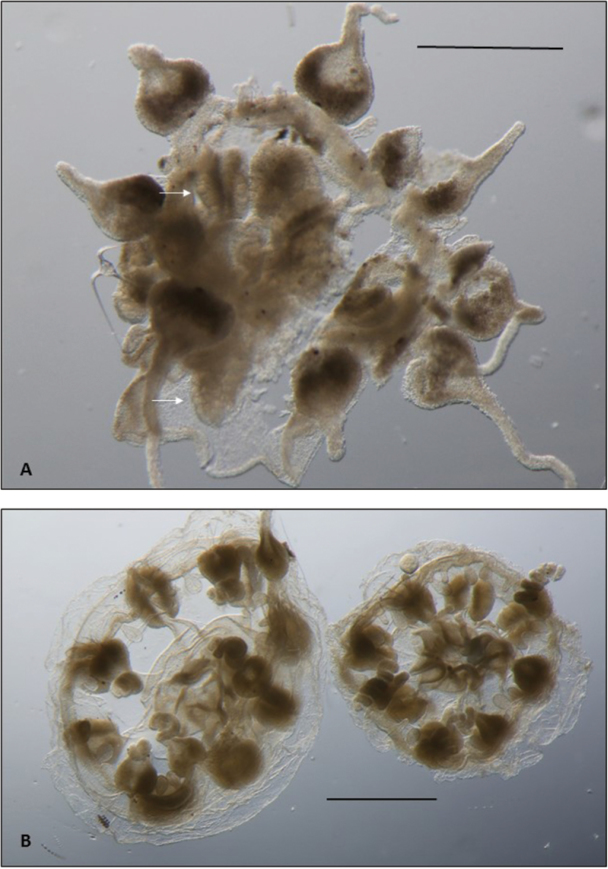
*Melicertissaantrichardsoni* sp. n. **A** Paratype QM G335901, female specimen; note ova on gonads (arrows) **B** Paratype lot of two, QM G335904. Scale bar: 1 mm (**A, B**).

***Paratypes***: QM G335904, Lot of two specimens, both male, BD ca. 2 mm, SD ca. 1 mm, hemi-gonad: 0.8 mm × 0.3 mm, tentacular bulbs seven, one clearly missing, base ca. 0.6 mm diameter (Figure 3B), Moreton Bay, Queensland, Australia, 27.35°S, 153.17°E, 1 m, (Study 1), coll. S. Pausina, 3 Dec 2010.

QM G335903, very poor condition, BD ca. 2 mm (Not figured), Moreton Bay, Queensland, Australia, 27.38°S, 153.39°E, 1 m, (Study 1), coll. S. Pausina, 19 Apr 2011.

**Figure 4.**
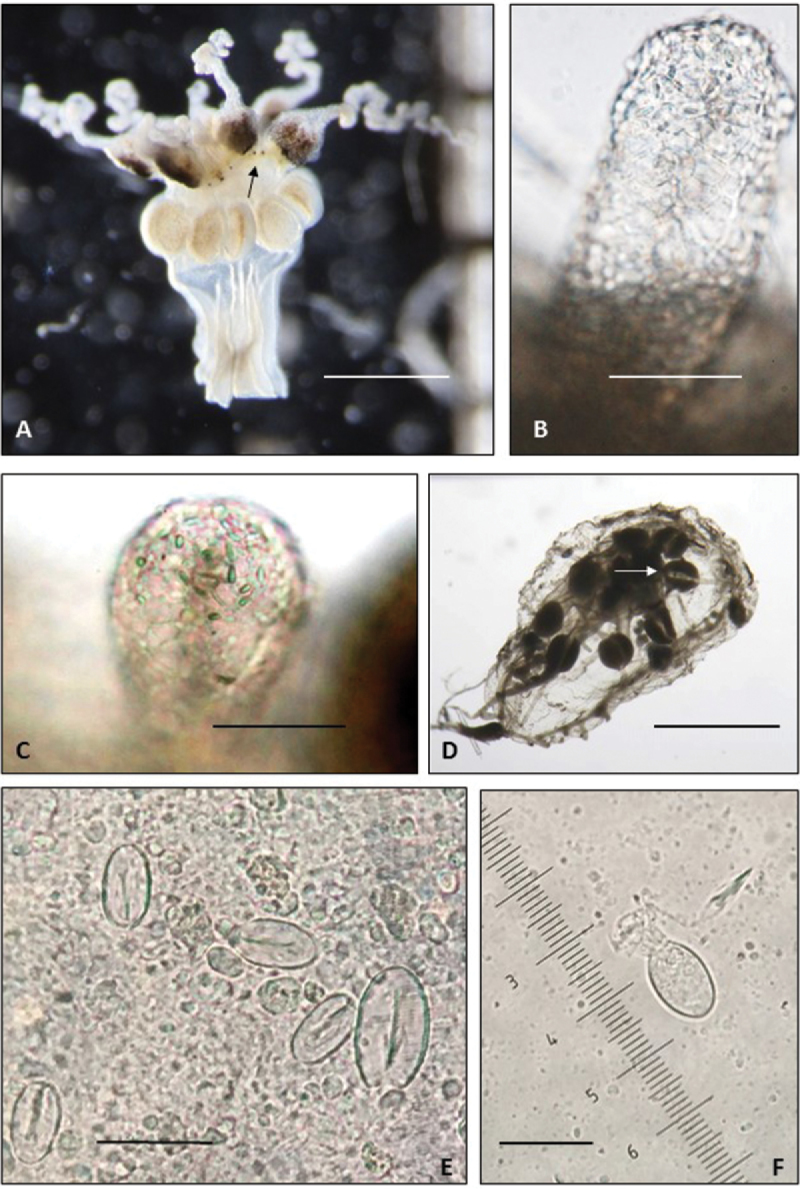
*Melicertissaantrichardsoni* sp. n. **A** Non-type specimen lost during study, inverted, note dark pigmenting of tentacle bulbs and ocelli (arrow) **B** Holotype QM G335899, cordylus, note cap-like position of nematocysts **C** Holotype QM G335899, cordylus, detail of nematocysts **D** Paratype QM G335902, note conspicuous radial canals (e.g., arrow) **E** tentacular nematocysts **F** nematocyst from whole body squash (QM G335905 and QM G335906 collectively). Scale bars: 1 mm (**A, D**); 50 µm (**B, C**); 20 µm (**E, F**).

QM G335902, Male, BD ca. 2.4 mm, hemi-gonad: 0.8-0.9 mm x 0.3 mm, tentacle bulbs seven (five well developed, plus two small ones), base ca. 0.6 mm (Figure 4D), Moreton Bay, Queensland, Australia, 27.16°S, 153.22°E (Study 2, (CSIRO MB55)), 1-2 m, coll. CSIRO, 27 May 2011.

QM G335900, Male, flattened specimen, BD ca. 2 mm. (Not figured); Moreton Bay, Queensland, Australia, 27.28°S, 153.08°E, (Study 2), (CSIRO MB74), 1-2 m, coll. CSIRO, 19 Dec 2011.

QM G335901, Female, flattened specimen, eigth tentacles, BD ca. 2 mm (Figure 3A), Rous Channel, Moreton Bay, Queensland, Australia, 27.29°S, 153.34°E, 1-2 m, (Study 3); coll. CSIRO 8 Apr 2014.

####### Other material.

QM G335905, QM G335906, microscope slide nematocyst preparations, Moreton Bay, Queensland, Australia, 27°S, 153°E, coll. CSIRO, 19/3/2011-20/5/2013 (Figure 4E, F).

####### Diagnosis.

*Melicertissa* with pairs of small oval hemi-gonads near the base of each radial canal; with eight tentacles and approximately two cordyli and one statocyst between successive tentacles; with an extremely small body size at maturity (ca. 2 mm).

####### Description of holotype.

Umbrella isosceles trapezoid-shaped in lateral view, aborally flattened, with the margin curving inwards at the edges, possibly due to preservation (Figure 2A, C). Velum well developed, encircling the inner side of the umbrellar margin, ca. 1/3 radius (Figure 2A).

Tentacles eight, six fully developed and two incompletely developed; hollow; coiled; more or less evenly located around the bell margin (Figure 2A–C). Tentacle bulbs voluminous, heart-shaped, short; approximately 0.3 mm across the base. Two ventral rows of nematocysts run parallel along the length of the tentacles (Figure 2B).

Cordyli club-shaped with a swollen end and slender stalk, almost half as long as the tentacle bulbs (Figure 2B), typically two between adjacent tentacles, ca. 200 μm long; with a nematocyst cap (Figure 4B, C).

Between adjacent cordyli typically lies another structure different in form, here interpreted as a statocyst. Compared to the tapered stalk of the cordylus, this structure is more evenly columnar or thimble-shaped with straight sides and a rounded distal end (Figure 2A, C). No cirri were observed associated with the tentacles or the umbrella margin.

Stomach amorphously round-ish, broad, nearly 1/2 BD in width (Figure 2A). Manubrium sculpted into eight vertical ridges, similar in appearance to a Greek column. Mouth shaped into eight smoothly rounded lobes with a simple margin (Figure 2A). In lateral view, the stomach occupies almost half the bell cavity, and the mouth nearly reaches the bell margin (Figure 2C).

Radial canals eight, relatively broad, straight-sided throughout length, clearly visible from the stomach to the ring canal (best illustrated in Figure 4D in paratype QM G335902).

Gonads in eight pairs of oval hemi-gonads straddling each radial canal adjacent to the stomach, occupying the proximal third of each radial canal (Figure 2A). Each hemi-gonad is voluminous with a smooth surface, interpreted to be male; approximately 230 μm long and half as wide.

Colour not noted in living specimen, but preserved specimen has a transparent bell with brown gonads, tentacle bulbs and stomach.

####### Type locality.

Moreton Bay, Queensland, Australia.

####### Variation from holotype.

One specimen (QM G335901) has lumpy gonads with obvious ova (Figure 3A), and is interpreted as a mature female. All other paratypes, which are nearly the same size, have smooth gonads and are interpreted as male.

Three of the specimens (two in lot QM G335904, and one in QM G335902) are each missing one tentacle bulb; these are not variants in merosity, but rather, they are octamerous with one bulb simply absent (Figure 3B). The holotype also has two tentacles in various stages of development, as one might expect following an injury (Figure 2A).

####### Colour.

Paratypes have a transparent and colourless bell and cordyli, pale yellowish gonads, and darkly pigmented tentacle bulbs. A black ocellus is found at the base of each tentacle and cordylus (Figure 4A).

####### Nematocysts.

*Melicertissaantrichardsoni* sp. n. has a cnidome of two size classes of oval cnidae on the tentacles (Figure 4E). There is only one size class of oval cnidae on the body (Figure 4F) and cordyli (Figure 4B, C).

On the tentacles, the larger size class is nearly 19 μm long by 10 μm wide, and bears a conspicuous barb on the free (= aboral) end of the undischarged shaft; we interpret this type as a stenotele. The smaller size class is approximately half the size of the larger, and bears a conspicuous v-shaped notch on the free (= aboral) end; we interpret this type as a microbasic *p*-mastigophore.

On the cordyli, nematocysts are scattered throughout the tip in a distal cap. The cordyli nematocysts are similar in shape but smaller in size (8 μm) than the small size class on the tentacles. However, we were unable to observe internal structures and were unable study them directly (Figure 4B, C).

####### Etymology.

The specific epithet, *antrichardsoni*, is given to honour Professor Anthony J. Richardson of CSIRO and the University of Queensland. Anthony has encouraged and supported the research of plankton from Moreton Bay and around Australia through the Australian Plankton Survey.

####### Environmental notes.

*Melicertissaantrichardsoni* has been found throughout the warmer months of the year from December to May, in salinities from 26–34 PSU and water temperatures from 18.84–26.5°C.

####### Systematic remarks.

*Melicertissaantrichardsoni* is the eighth species in the genus (Table 1). Its gonads make it entirely unique within the genus, as these organs in other species are linear and either tapering, sinuous, or foliaceous, whereas in *M.antrichardsoni* they are small and oval in tight pairs. Another distinguishing feature is that *M.antrichardsoni* is much smaller than its congeners, being less than 30% the size of the other smaller species, *M.mayeri*Kramp (1959) and *M.platygastra*Nair (1951).

**Table 1.** Summary of main features from each one of the known species of *Melicertissa*. Data from original species descriptions along with Mayer (1910) and Kramp (1961a). Abbreviations: bell diameter (BD), radial canals (RC).

Species	BD	Bell shape	Stomach	Mouth	Gonads	Tentacles	Cordyli	Ocelli	Cirri	Locality
*M.adriatica* Neppi, 1915	46 mm	Flatter than hemisphere, with thick jelly	Short	8 short crenulated lips	Linear along length of RC	24	3–5 between adjacent tentacles	On each cordylus	More # than cordyli	Adriatic sea
*M.clavigera* Haeckel, 1879	10 mm	Flat to hemispherical; jelly thin	Flat, or in a long narrow manubrium	Quadratic, drawn into 4 or 8 short lips	Foliaceous along most of RC, thicker in middle half	8, with thick conical base	3 between adjacent tentacles	On tentacles and cordyli	(Not indicated)	Canary Islands
*M.malayica* (Maas, 1905)	32 mm	Flat	Very flat	8-lobed	Linear on proximal 1/3 of RC, slender, tapering	>160, with slight bulbous swelling	At irregular intervals between tentacles	~ 5–6 per octant at base of some tentacles	Sparser than cordyli	Malay Archipelago
*M.mayeri* Kramp, 1959	7 mm	Flatter than hemisphere; with moderately thin walls	Flat	8 short, simple lips	Somewhat sinuous, upon the middle half of RC	16, with long, hollow, tapered bulbs	16	32	(Not indicated)	Tortugas, Florida
*M.orientalis* Kramp, 1961b	11 mm	Flatter than hemisphere; fairly thick jelly	Flat and broad	8 faintly indicated lips with a smooth margin	Wavy, lateral bands with about 5 extensions, on distal 2/5 of RC	17, with heart-shaped bulb	1–3, mostly 2, between adjacent tentacles	Black, on base of tentacles and cordyli	(Not indicated)	Green Island, Great Barrier Reef
*M.platygastra* Nair, 1951	7 mm	Watch-glass shaped, very thick in centre	Flat, half as wide as BD	8 lanceolate lips with thick wavy margins	Linear on outer half of RC in 3 continuous lumps	8, short stumpy, with large conical bulbs	4–6 in each octant	12–14 dark ocelli per octant	(Not indicated)	Trivandrum coast, India
*M.rosea* Bouillon, 1984	10 mm	(Not indicated)	1/3 BD	Formed into 8 folds	Foliaceous, almost as long as RC	40	40	At base of tentacles and cordyli	(Not indicated)	Papua New Guinea
*M.antrichardsoni* sp. n. Uribe-Palomino & Gershwin	2 mm	Isosceles trapezoid	Flat and broad, about half BD	8-lobed, with smoothly-rounded lips	8 pairs of oval hemi-gonads close to stomach	8, with heart-shaped bulbs	~ 2 between tentacles, plus a statocyst between	Black, at base of tentacles and cordyli	Not observed	Moreton Bay, Queensland

Compared to the other species with eight tentacles, namely *M.clavigera*Haeckel (1879) and *M.platygastra*Nair (1951), there are ample differences to separate *M.antrichardsoni*. Firstly, the tentacle bulbs of *M.clavigera* and *M.platygastra* are both thick and conical, whereas those of *M.antrichardsoni* are so bulbous as to be heart-shaped. Secondly, the lips in *M.platygastra* are lanceolate while those of *M.clavigera* are said to be quadratic, whereas these structures in *M.antrichardsoni* sp. n. are more smoothly rounded as one might expect an eight leafed clover to look.

Finally, *Melicertissaantrichardsoni* would be unlikely to be confused with *M.malayica*Maas (1905), the only other species with gonads adjacent to the stomach: in the latter the gonads are slender and tapered, there are more than 160 tentacles, and the cordyli are irregular, whereas in the former the gonads are oval, there are eight tentacles, and the cordyli are quite regular.

One may wonder about the relationship between *M.orientalis*Kramp (1961b) and *M.antrichardsoni*, with both being apparently endemic to Queensland. Both species have heart-shaped tentacle bulbs and nematocyst-studded cordyli however, they are remarkably dissimilar in their tentacles, gonads, and distribution. For example, *M.orientalis* from tropical waters of the Great Barrier Reef has seventeen tentacles and the gonads are in wavy bands along the distal 2/5 of the radial canals, whereas *M.antrichardsoni* from coastal, shallow, sub-tropical waters of southeast Queensland has only half as many tentacles and the gonads are in paired ovals adjacent to the stomach.

With the specimens of *M.antrichardsoni* at only two millimetres in diameter, it is logical to ask whether they might be juveniles. However, this is unlikely as the female specimen (QM G335901) appears to have mature ova and is near the same size as the other specimens. Specimens have been found on numerous occasions throughout the summertime over a period of five years.

Curiously, Munro (1940) mentioned finding specimens of ‘*Melicertiasa*’ at an area of Moreton Bay locally known as Waterloo Bay, at a sampling station located halfway between Manly and Green Island (not to be confused with Manly in New South Wales or Green Island in the Great Barrier Reef). He gave no other indication as to the identity of his specimens, but we wonder whether these might be the same species as ours. Currently the only species of *Melicertissa* known from Australia are *M.orientalis* and *M.antrichardsoni*.

We recognise *Melicertissaadriatica*Neppi (1915) with some caution. Kramp (1961a) considered *M.adriatica* to be in the Laodiceidae (p. 143) but noted in the addendum (p. 444) that Picard referred this species to *Octogonademediterranea*Zoja (1896) in the Mitrocomidae (Kramp 1961a, p. 157). In a subsequent publication, Kramp (1961b) elaborated, noting: “Dr. J. Picard (Marseilles) has informed me in a letter that *Melicertissaadriatica* Neppi is identical with *Octogonademediterranea* Zoja.” However, Zoja’s original illustration of *O.mediterranea* gives no indication of cordyli, which appear to have been clearly described in *M.adriatica* by Neppi, who differentiated them from both tentacles and cirri: “Zwischen je zwei Tentakeln drei bis fünf Randkolben mit einem schwarzen Ocellus und noch zahlreichere Cirren.” *Octogonade* has since been moved to the Tiaropsidae (Boero et al. 1987) on the basis of having compound statocysts. Bouillon and Boero (2000) and Bouillon et al. (2004) upheld *M.adriatica* as a laodiceid (with three types of marginal appendages, namely tentacles, cordyli, and cirri), whereas *Octogonade* has two types of tentacles and neither cordyli or cirri. Schuchert (2016), however, considered *M.adriatica* to be a junior synonym of *Octogonade*. Here, we consider *M.adriatica* to be a laodiceid based on its possession of three types of marginal appendages, whereas *Octogonade* has only two.

#### Family LOVENELLIDAE Russell, 1953

##### Genus *Paralovenia* Bouillon, 1984

###### Paralovenia yongalensis

Taxon classificationAnimaliaLeptothecataLaodiceidae

Gershwin & Uribe-Palomino sp. n.

http://zoobank.org/583DB2A1-6F80-4871-BEB2-DD2F3896295C

**Figure 5.**
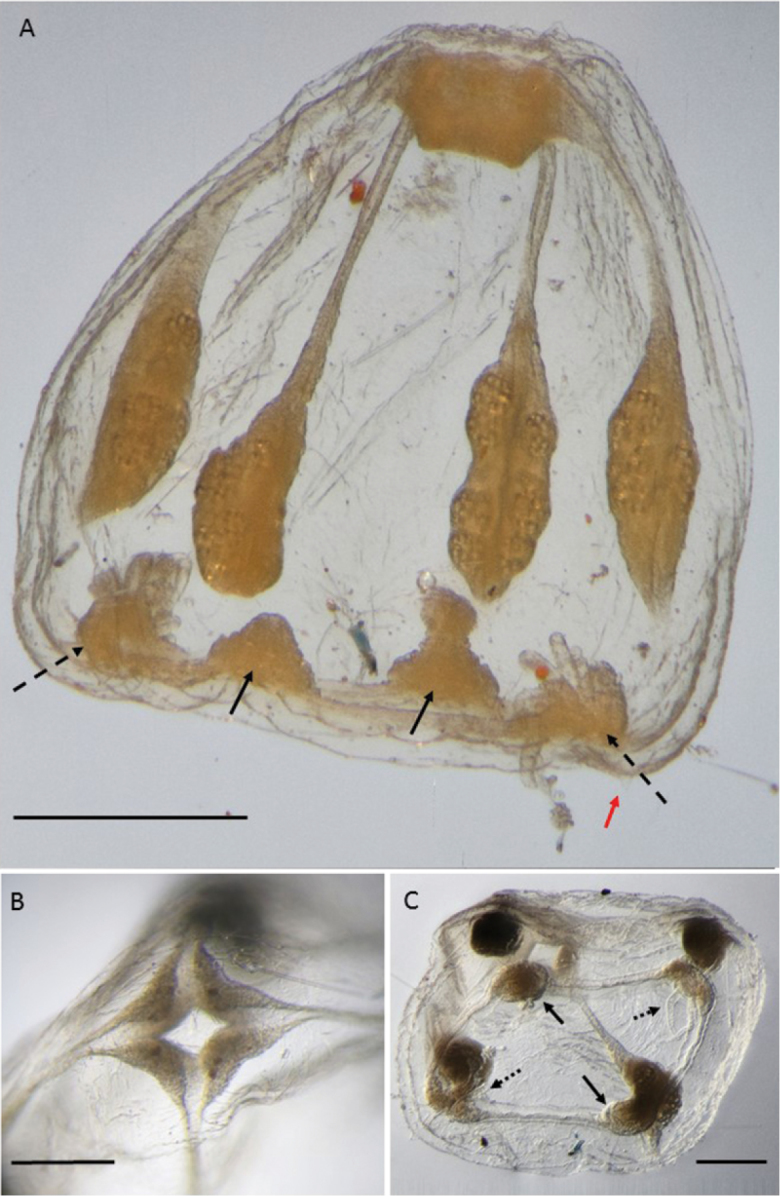
*Paraloveniayongalensis* sp. n., Holotype QM G335907. **A** habitus; note conical statocyst-like structure (indicated by red arrow) **B** Aboral view of stomach (cruciform structure closest to the viewer) and mouth (quadratic structure away from the viewer) **C** Oral view of margin (note that the gonads are the darkest brown structures, and the mouth is the quadratic structure near the upper left). Black arrows in **A** and **C**: tentacular bulbs indicated by solid arrows and bulbs bearing cirri indicated by dashed arrows. Scale bars: 0.5 mm (**A**); 250 µm (**B, C**).

**Figure 6.**
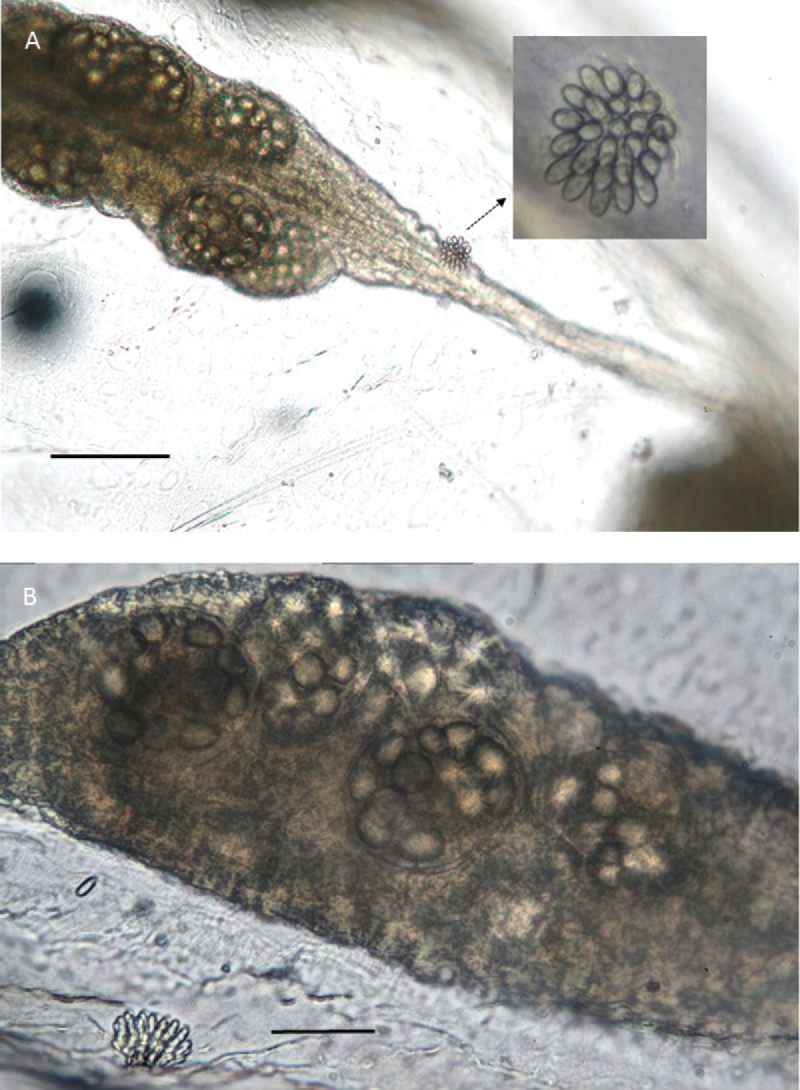
*Paraloveniayongalensis* sp. n., Holotype QM G335907. **A** Gonad with egg follicles (upper left; 2^nd^ right gonad in habitus photo in Figure 5A), a subumbrellar nematocyst cluster can be seen near the centre, and is detailed in the box to the right **B** Another gonad with egg follicles (far right gonad in habitus photo in Figure 5A), and a subumbrellar nematocyst cluster to the lower left. Scale bars: 90 µm (**A**); 40 µm (**B**).

**Figure 7.**
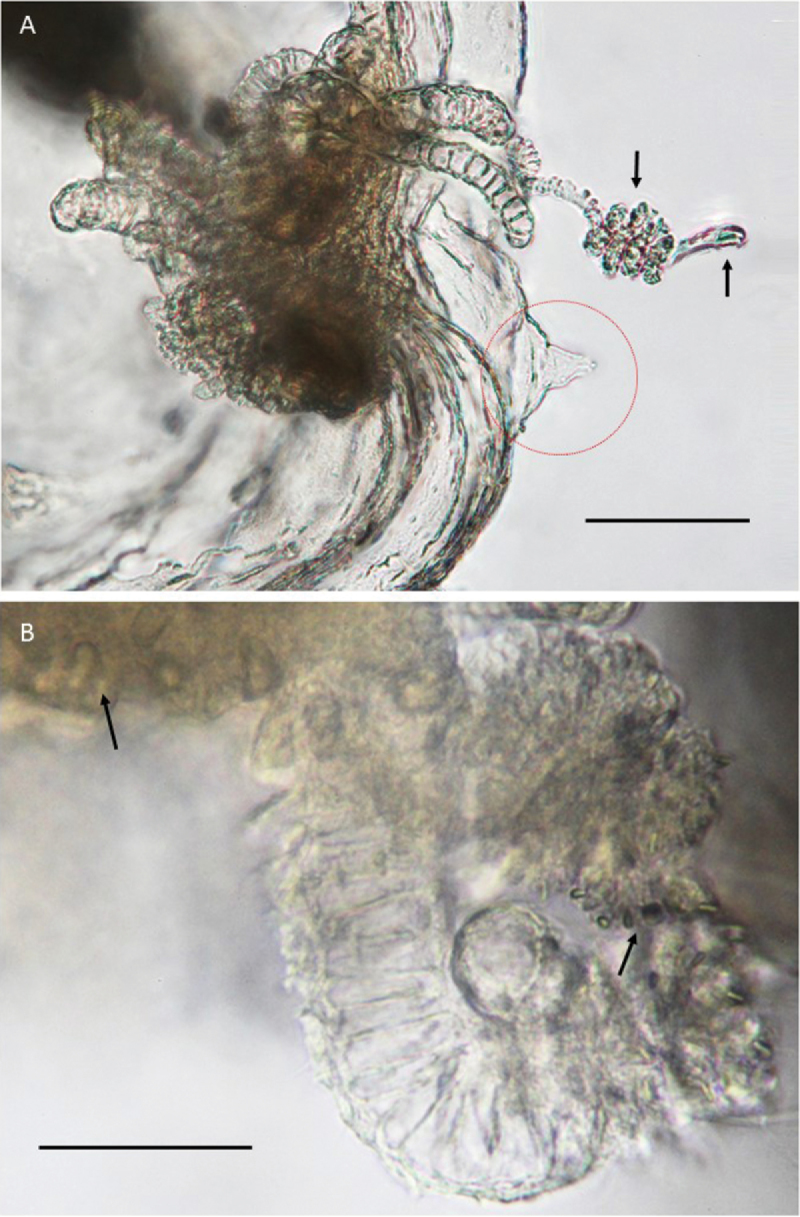
*Paraloveniayongalensis* sp. n., Holotype QM G335907. **A** Magnified view of cirri cluster; note statocyst-like structure (red circle) and two sizes of nematocysts on coiled cirrus (arrows) **B** Detail of nematocysts in bulb (upper left arrow) and cirrus (lower right arrow). Scale bars: 100 µm (**A**); 50 µm (**B**).

**Figure 8.**
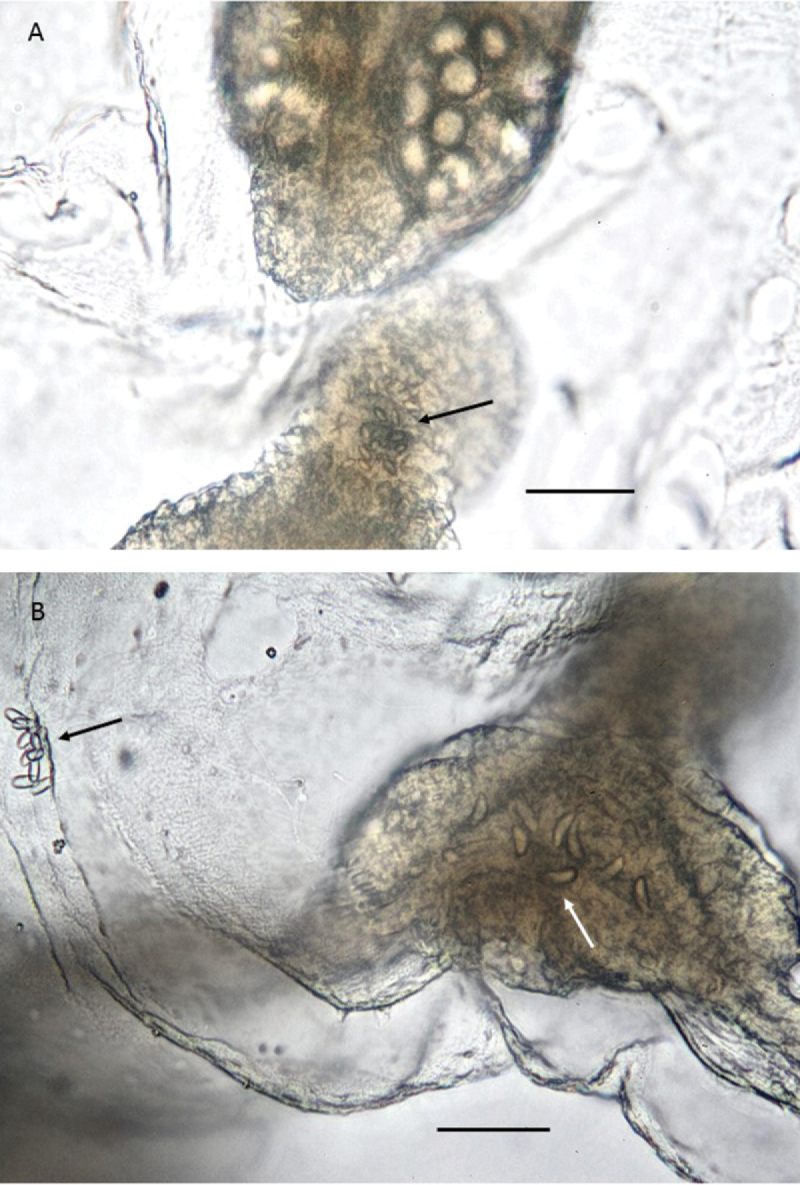
*Paraloveniayongalensis* sp. n., Holotype QM G335907. **A** Detail of nematocysts of the tentacle (arrow) **B** Detail of nematocysts of the tentacular bulb (white arrow) and of a subumbrellar nematocyst cluster (black arrow). Scale bars: 50 µm (**A, B**).

Figures 5 , 6 , 7 , 8

####### Type material.

***Holotype***: QM G335907. Female, BD ca. 1.47 mm, tentacular bulbs two and two non-tentacular clusters of cirri (Figures 5A, C); near *SS Yongala* shipwreck, Queensland, Australia, 19.31°S, 147.62°E, 0–28 m, drop net; coll. IMOS-CSIRO, 27 Sep 2016.

####### Diagnosis.

*Paralovenia* with a relatively short, bell-shaped body with a truncated apex; with well-developed spindle-shaped gonads in the distal half of radial canals, not reaching the margin; with two opposite perradial triangular bulbs bearing tentacles, and two similar opposite perradial bulbs each bearing approximately 10–12 cirri; with open statocysts on a broad conical base; nematocysts arranged in flat roundish clusters on the subumbrellar surface.

####### Description of holotype.

Umbrella bell- to barrel-shaped, broadest about 2/3 of the way down, truncate to slightly indented aborally (Figure 5A); sparsely scattered with minute nematocyst clusters (Figure 6). Oral margin curving slightly inwards possibly due to preservation. Velum narrow.

Tentacles two, opposite, on voluminous, triangular perradial basal bulbs, approximately 250 μm across the base (Figures 5A, C). The tentacles are tightly contracted. The other two perradial bulbs are considerably smaller, and each bears 10–12 solid, straight, thick cirri approximately 300 μm long and decorated with a continuous spiral pattern or a series of fine rings along the whole length (Figures 5A, 7A). One of the cirri in the preserved specimen is coiled and ends in two large, long bean-shaped nematocysts (Figure 7A); the extent to which this coiling is normal in life is unknown.

Other marginal structures: opposite the radial canal associated with one of the tentacle bulbs bearing cirri, there exists a small gelatinous “thorn” projecting outward (Figures 5A, 7A). Under high magnification, this conical structure appears to have a central canal and ends in what appears to be an open statocyst (Figure 7A). Whether this structure exists on the other perradii could not be determined. No ocelli observed.

Stomach small, cruciform at base (Figure 5B), approximately 300 μm in diagonal width, with a short broad tapering manubrium, square in cross section (136 μm long), lacking a gastric peduncle (Figure 5A). Mouth simple without lips, perfectly quadrangular (Figure 5B).

Radial canals four, straight sided throughout length clearly visible from the stomach to the ring canal, approximately 30 μm in diameter and expanded proximally to create mesentery-like connections with the stomach (Figure 5A). Ring canal also straight-sided and of a similar width (Figure 5C).

Gonads four, spindle-shaped, starting approximately half way from the stomach toward the margin, covering 2/5 of the entire length of the radial canal, absent on the distal fifth of the radial canal (Figure 5A). Eggs, in clusters on lumpy follicles 80–90 μm in diameter; easily identified in each of the gonads (Figures 5A, 6).

####### Type locality.

Near the shipwreck *SS Yongala*, south-east of Townsville, Queensland, Australia.

####### Nematocysts.

No nematocyst preparation was made because the only available specimen is the holotype and it is too small to take sections from without destroying it. Therefore, the following description of nematocysts is based on in situ observation only. Bell nematocysts are mostly in roughly circular clusters up to 30 μm in diameter on the subumbrellar surface. Each cluster consists of 4–20 nematocysts. The nematocysts are oval in shape and approximately 12 μm long and half as wide (Figure 6). Tentacular bulbs: containing bean-shaped nematocysts approximately 15 μm long (Figure 8B). Tentacles: nematocysts are oval shape and they are approximately 7.5 µm long (Figure 8A). Cirri bulbs: the same type of nematocysts were found in the cirri bulbs as those found in the tentacular bulbs (Figure 7B). Cirri: nematocysts were not observed in the majority of the cirri. However, in the coiled cirrus (explained above), the coiled region contained numerous very small nematocysts (ca. 3 µm long) and the terminal end contained two bean-shaped nematocysts approximately four times the size of the other type found more proximally (Figure 7).

####### Etymology.

The specific name *yongalensis* is given honouring the area where people lost their lives on board the *SS Yongala* that sank not far away from Cape Bowling Green, Queensland, in 1911. This area is a popular scuba diving site and it is also the location of one of the IMOS National Reference Stations. This is not to be confused with the town from South Australia of the same name.

####### Environmental notes.

The specimen was collected in a vertical drop net from the surface to 28 m in an area of maximum 30 m depth; we therefore do not know its exact location in the water column. The sea surface temperature at the time was 27.5 °C.

####### Systematic remarks.

Like its congeners, *Paraloveniayongalensis* has two opposite tentacles and two opposite clusters of short cirri. However, the gonad shape and position immediately distinguish it from the others (Table 2).

Whereas *P.bitentaculata*Bouillon (1984) and *P.latigaster* Xu & Huang (2004) both have a deep pyriform bell and long cylindrical gonads, they are distinguished primarily on the number of cirri on each marginal cluster, about six in the former and twelve in the latter, and on the size of the stomach, short in the former and huge in the latter.

**Table 2.** Summary of main features from each of the known species of *Paralovenia*. Data from original species descriptions. Abbreviations: bell height (BH), radial canals (RC).

Species	BH	Body shape	Stomach	Mouth	Gonads	Tentacles	Cirri	Sense organs	Locality
* P. bitentaculata * Bouillon (1984)	1.4 mm	Deep bell, piriform	Short	4 lips, with a quadrangular manubrium	Voluminous, cylindrical, occupying central ¾ of RC	2 opposing, on large, conical, bulbous bases	2 opposing clusters of 6, on bulbs smaller than those bearing tentacles	Statocysts and ocelli absent	Papua New Guinea
*P.latigaster* Xu & Huang (2004)	~ 2 mm	Deep bell, piriform	Large, broad	Not described	Voluminous, cylindrical, extending along each RC from the stomach nearly to the margin	2 opposing, on large, conical, bulbous bases	2 opposing clusters of up to 12	Statocysts absent; ocelli not specified	Taiwan Strait, China
*P.yongalensis* sp. n. Gershwin & Uribe-Palomino	1.4 mm	Short bell, truncated apex; with subumbrellar nematocyst clusters	Short, cruciform	Mouth perfectly quadrangular, with a short quadrangular manubrium	Voluminous, spindle-shaped, in distal half of RC, well separated from the margin	2 opposing, with large broad bulbs	2 opposing clusters of 10–12 solid straight cirri	Ocelli absent; statocysts open, on broad conical base	Great Barrier Reef, Australia

The cirri of *P.yongalensis* are more similar in number to *P.latigaster*, while the stomach is small, like that of *P.bitentaculata*; *P.yongalensis* differs from both in having a relatively shorter, rounder body, and shorter, more spindle-shaped gonads, tapered at both ends. Moreover, *P.yongalensis* is approximately the same size as *P.bitentaculata*.

The cirri are worthy of discussion. Bouillon (1984) described and illustrated the cirri in *P.bitentaculata* as spiralled in form, while Xu and Huang (2004) do not describe in detail the cirri of *P.latigaster*. In *P.yongalensis*, the cirri are straight, solid, relatively thick, with a rounded end, with a series of external rings or a continuous ornamentation spiralling along their length. However, one of the cirri was tightly coiled, so we are unsure whether this coiling is the normal state in life for all the cirri, or for just one in each cluster, or if it was an artefact of preservation.

Interestingly, *P.yongalensis* has a few flat round clusters of nematocysts on the subumbrellar surface. Neither Bouillon (1984) nor Xu and Huang (2004) mentioned finding this characteristic in the other two species of *Paralovenia*.

Similarly, the conical statocyst-like structure in *P.yongalensis* is very interesting to us. Bouillon (1984) stated for *P.bitentaculata*, “Nous n’avons pas observé d’organes des sens”; we interpret this to mean that he found neither statocysts nor ocelli. However, Xu and Huang (2004) specifically stated that *P.latigaster* was “without statocysts”. The structure that we have observed is extremely small and could be overlooked, but we doubt that all three researchers would have done so. Perhaps more intriguing to us is the fact that open statocysts are typically more “finger-shaped” (e.g., Russell 1953: 13, text fig. 7C), so besides being apparently unique in the genus, this also is a form of statocyst that we have not previously seen.

We recognise the value of providing DNA sequences as molecular evidence to support the description of new species when it is practical. In the present case, all the specimens were preserved in formalin, making successful extraction of DNA unlikely. Moreover, with so few specimens of *Melicertissa* and only one specimen of *Paralovenia*, we consider the morphological approach to be the less-destructive method to characterise these two new species.

Finally, while we believe that the fully-developed gonads for both species suggest that they are mature specimens, this hypothesis may be tested using DNA sequencing in future research.

## Supplementary Material

XML Treatment for Melicertissa antrichardsoni

XML Treatment for Paralovenia yongalensis
